# Direct Determination of Pyrazinamide (PZA) Susceptibility by Sputum Microscopic Observation Drug Susceptibility (MODS) Culture at Neutral pH: the MODS-PZA Assay

**DOI:** 10.1128/JCM.01165-19

**Published:** 2020-04-23

**Authors:** Roberto Alcántara, Patricia Fuentes, Lisette Marin, Daniela E. Kirwan, Robert H. Gilman, Mirko Zimic, Patricia Sheen

**Affiliations:** a Laboratorio de Bioinformática y Biología Molecular, Facultad de Ciencias y Filosofía, Universidad Peruana Cayetano Heredia, Lima, Perú; b Infection and Immunity Research Institute, St George’s, University of London, London, United Kingdom; c Department of International Health, Johns Hopkins Bloomberg School of Public Health, Baltimore, Maryland, USA; Carter BloodCare and Baylor University Medical Center

**Keywords:** drug susceptibility, MODS, pyrazinamide, sputum, tuberculosis

## Abstract

Pyrazinamide (PZA) is considered the pivot drug in all tuberculosis treatment regimens due to its particular action on the persistent forms of Mycobacterium tuberculosis. However, no drug susceptibility test (DST) is considered sufficiently reliable for routine application. Although molecular tests are endorsed, their application is limited to known PZA resistance associated mutations. Microbiological DSTs for PZA have been restricted by technical limitations, especially the necessity for an acidic pH. Here, for the first time, MODS culture at neutral pH was evaluated using high PZA concentrations (400 and 800 μg/ml) to determine PZA susceptibility directly from sputum samples. Sputum samples were cultured with PZA for up to 21 days at 37°C. Plate reading was performed at two time points: R1 (mean, 10 days) and R2 (mean, 13 days) for each PZA concentration. A consensus reference test, composed of MGIT-PZA, *pncA* sequencing, and the classic Wayne test, was used. A total of 182 samples were evaluated. The sensitivity and specificity for 400 μg/ml ranged from 76.9 to 89.7 and from 93.0 to 97.9%, respectively, and for 800 μg/ml ranged from 71.8 to 82.1 and from 95.8 to 98.6%, respectively. Compared to MGIT-PZA, our test showed a similar turnaround time (medians of 10 and 12 days for PZA-sensitive and -resistant isolates, respectively). In conclusion, MODS-PZA is presented as a fast, simple, and low-cost DST that could complement the MODS assay to evaluate resistance to the principal first-line antituberculosis drugs. Further optimization of test conditions would be useful in order to increase its performance.

## Abstract (precis)

Pyrazinamide (PZA) is considered the pivot drug in all tuberculosis treatment regimens due to its particular action on the persistent forms of Mycobacterium tuberculosis. However, no drug susceptibility test (DST) is considered sufficiently reliable for routine application. Although molecular tests are endorsed, their application is limited to known PZA resistance associated mutations. Microbiological DSTs for PZA have been restricted by technical limitations, especially the necessity for an acidic pH.

## INTRODUCTION

According to the World Health Organization (WHO), tuberculosis (TB) is one of the top ten causes of death worldwide and the most important cause by a single infectious agent (1). In 2017 there were 10 million new cases of disease caused by Mycobacterium tuberculosis and over 1.3 million deaths (1). Currently, drug resistance is the main factor that impedes TB eradication initiatives. The capacity to detect drug-resistant TB remains limited in low- and middle-income countries, where it is most needed (2). Discovered in 1953, pyrazinamide (PZA) has proven to be an indispensable first-line anti-TB agent (3–5) which, unlike other first-line agents, exhibits a unique sterilizing ability, inhibiting slowly metabolizing or nonmetabolizing bacilli (6, 7). Its inclusion in treatment regimens enabled them to be shortened from 9 to 6 months (8).

Despite this, its mechanism of action remains poorly understood (9). It has been shown that PZA is converted to its active component, pyrazinoic acid (POA), by the pyrazinamidase enzyme (PZAse) in the intracellular environment of M. tuberculosis. It is accepted that POA causes cytoplasmic acidification and disruption of membrane potential (10–12) and possibly interacts with a range of intracellular targets (7, 13). Mutations in *pncA*, the gene encoding PZAse, have been reported as the primary cause of PZA resistance (7, 14). It has been estimated that between 40 and 80% of multidrug-resistant TB (MDR-TB) and 100% of extensively drug-resistant TB (XDR-TB) strains have also been reported as resistant to PZA (5, 15–17). Even though PZA is considered the only first-line drug to be preserved in all future treatment regimens (18–21), PZA DST is not routinely performed and is seldom used to plan a personalized treatment regimen (9).

The development of molecular diagnostic tests to detect PZA resistance have been limited due to the large number of mutations along the *pncA* gene (14), the lack of a mutation hot spot, and the fact that not all mutations cause PZAse impairment or have been associated with a specific PZA susceptibility phenotype (15, 22–25). However, recently some genotypic DST have been reported with considerable sensitivity and specificity, such the Genoscholar PZA-TB II LPA (Nipro Corporation, Japan), which is a line probe assay (LPA), and a high-resolution melting assay. Previous studies have reported an agreement of 97.6% between the PZA-TB II LPA and a composite reference (*pncA* sequencing plus phenotypic DST) when heteroresistance is excluded (26), and a sensitivity of 93.2% and a specificity of 91.2% when the test was compared to only *pncA* sequencing (27). On the other hand, the high-resolution melting assay has been described as having an agreement of 94 and 84% with *pncA* sequencing and MGIT-PZA, respectively (28). Nonetheless, one limitation for the immediate application of these tests is that the genetic diversity of *pncA* mutations can vary appreciably between different geographical regions, affecting the sensitivity and specificity of the test (26, 27). As a matter of fact, the correlation between *pncA* sequencing and detection of mutations with PZA resistance varies among countries low and high endemicities, with reported estimates of 41% in Taiwan, 67% in South Africa, 72 to 84% in Brazil, 91% in China, and 97% in China and Japan (7, 18).

There is currently no WHO-endorsed phenotypic drug susceptibility test (DST) for detecting PZA resistance due to various technical limitations affecting the utility of currently available assays. These limitations include long turnaround times, poor reproducibility, requirements for an acidic pH, and medium alkalization due to inoculum size (9, 15, 29, 30). Performing direct phenotypic DST for PZA has been hampered by the difficulty in demonstrating anti-TB activity of the drug when analyzed under conventional culture conditions. The widely accepted explanation for this is the requirement of an acidic environment for PZA to exert anti-TB activity versus the pH close to neutral of the culture medium (9, 10, 18, 31–33). At present, the Bactec MGIT 960 PZA (MGIT-PZA) assay is the accepted standardized phenotypic DST (34). However, several publications have reported a high rate of false PZA resistance and poor reproducibility, both drawbacks related to pH variation produced by the inoculum size used in the assay (35–39).

The microscopic observation drug susceptibility (MODS) assay is a relatively rapid and inexpensive direct drug susceptibility test used in the diagnosis of TB and MDR-TB (40–42) and XDR-TB (43–45). Previous studies have evaluated the use of MODS culture for analysis of PZA resistance using PZA concentrations ranging between 6.25 and 3,200 μg/ml (46–48). These studies have compared MODS for PZA susceptibility determination against the MGIT-PZA (46), the proportion method (47), and the absolute concentration method (48), reporting sensitivity and specificity ranges of 95.5 to 100% and 93.3 to 100%, respectively. In all cases, a high concordance between MODS and the selected reference method was observed, although all tests were performed with cultured M. tuberculosis strains and not directly from clinical sputum samples.

In the present study, for the first time, we evaluated the use of MODS to determine PZA resistance directly from sputum samples. MODS-PZA is a test that was conceived as a complement for the traditional MODS test to determine resistance to all the first-line antituberculosis drugs. MODS-PZA has been standardized based on the fact that several studies have reported that stress factors can strengthen PZA activity even in nonacidic media (6, 9, 31, 49–51). These stress factors must affect the metabolism of M. tuberculosis in order to predispose the mycobacteria to show a PZA-sensitive phenotype (9). This is in keeping with observations that PZA performs better against M. tuberculosis bacilli with a reduced metabolic rate compared to those in log-phase growth (6, 49). Therefore, for this study, it was assumed that the normal decontamination step might produce a degree of stress that can nonspecifically reduce the mycobacterial metabolic rate. We performed MODS culture directly from sputum samples using high drug concentrations (400 and 800 μg/ml) and a pH close to neutral (pH 6.8). These results are compared to a consensus reference test based on three tests: MGIT-PZA, the classical Wayne test, and *pncA* sequencing.

## MATERIALS AND METHODS

### Sample selection.

Sputum samples were obtained from patients enrolled in the TB programs at the Hospital Cayetano Heredia and Hospital Nacional Dos de Mayo, both in Lima, Peru, and the Regional Tuberculosis Reference Laboratory (Callao Reference Laboratory), Callao, Lima, Peru. All selected samples had a minimum volume of 2 ml and were determined to be positive for M. tuberculosis by MODS culture. Since an estimated prevalence of 50% of PZA resistance has been reported for MDR-TB strains (5, 15), ca. 50% of the selected samples were MDR-TB or monoresistant to isoniazid (INH) or rifampin (RIF) and 50% were INH and RIF susceptible in order to obtain an adequate number of PZA-resistant strains. Data regarding sex, age, and TB treatment were collected when available. Ethical approval was obtained from the ethical committee from the Universidad Peruana Cayetano Heredia (IRB 418-20-15).

### MODS for *M. tuberculosis* identification and MDR-TB detection.

Sputum samples (2 ml) were decontaminated according to the standardized MODS protocol (52). Briefly, the sputum sample was mixed with 2 ml of NaCl-NaOH and incubated for 15 min at room temperature. Phosphate buffer (pH 6.8) was then added to neutralize the sample, which was then centrifuged for 15 min at 3,000 × *g*. The decontaminated pellets were resuspended in 6 ml of Middlebrook 7H9 (Fisher Scientific) medium enriched with oleic acid-albumin-dextrose-catalase (OADC) and polymyxin B, amphotericin B, nalidixic acid, trimethoprim, and azlocillin (PANTA). MODS testing was performed in 24-well plates according to the standardized MODS protocol (52). Briefly, 100 μl of 7H9 medium was dispensed into the first and second wells (growth control wells), and 100 μl of drug suspension (final concentrations, INH at 4 μg/ml and RIF at 10 μg/ml) was added to the third and fourth wells, respectively (drug susceptibility wells). Next, 900 μl of the resuspended pellet was dispensed into these four wells to make a total volume of 1 ml. The plate was incubated at 37°C for a maximum of 21 days. A plate reading was performed using an inverted microscope at 100× total magnification in order to observe the cording growth of M. tuberculosis (52). Both control wells were evaluated at day 5 and then every other day. Positive growth was reported when at least two CFU were observed. Drug susceptibility wells (INH and RIF) were evaluated at the time that growth was observed in both control wells. Drug resistance was reported when positive growth was observed in these wells.

### MODS for PZA susceptibility determination (MODS-PZA).

The goal of sample decontamination prior to culture is to drastically reduce the number of viable contaminants, but the reagents used in this process also affect the viability of M. tuberculosis (53–56). Therefore, we hypothesized that the decontamination step could produce some degree of stress that permits PZA to work. Consequently, MODS-PZA (MODS for PZA susceptibility determination) was standardized to determine PZA susceptibility using 7H9 medium enriched with OADC-PANTA directly from sputum samples at a pH close to neutral.

**FIG 1:**
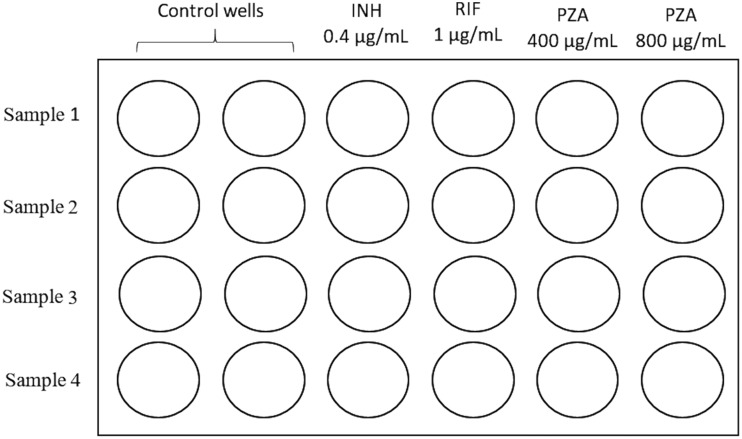
MODS-PZA plate setting. Four samples were evaluated in each plate. Two control wells were included for each sample.

**FIG 2:**
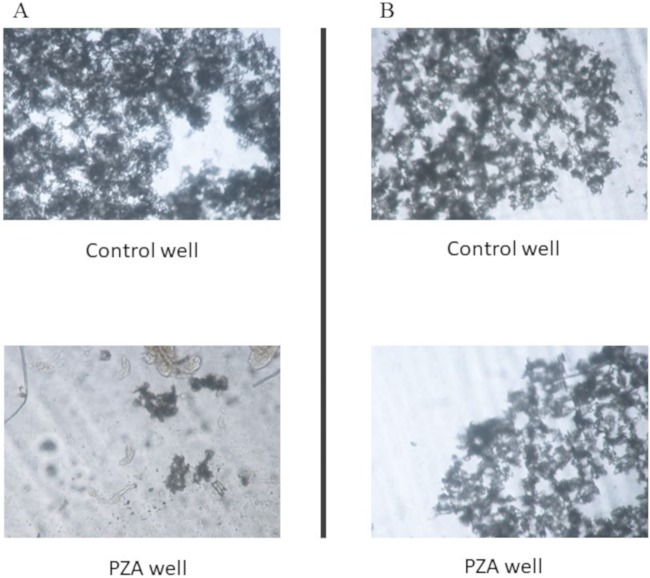
MODS-PZA results. PZA susceptibility determination was performed by inverted microscopy. Both control and drug wells were evaluated. (A) PZA-resistant isolate. Bacterial growth is observed in both control and PZA wells. (B) PZA-susceptible isolate. Bacterial growth is strongly differentiated between control and PZA wells.

From each decontaminated sample, 900 μl was inoculated into the fifth and sixth wells of the same culture plate for MDR detection. Next, 100 μl of 4,000 and 8,000 μg/ml PZA was added to the fifth and the sixth wells (PZA wells), respectively (Fig. 1). The plate was read as previously mentioned for MDR-TB detection (first plate reading [R1]). PZA resistance was reported when a growth level similar to that in the control well was observed (Fig. 2A). Alternatively, PZA susceptibility was reported when no growth or a lower growth level than in the control well was detected (Fig. 2B). Samples that did not show any growth were incubated for 21 days and evaluated every other day. A second plate reading (R2) was reported if growth was observed before 21 days of culture, following the previous criteria to determine PZA susceptibility.

### Consensus reference test for PZA susceptibility determination.

Currently, there is no single test that is recognized as a reference method to determine PZA susceptibility. Consensus reference standards have been reported as an option for evaluating drug susceptibility when no other reference method is available (57). In this study, Bactec MGIT 960 PZA (MGIT-PZA), the classic Wayne assay, and *pncA* sequencing to detect nonsynonymous mutations were combined into a consensus reference test (CRT). PZA susceptibility was reported when a susceptible result was obtained in at least two of the three methods; conversely, PZA resistance was reported when a resistant result was observed in at least two of the three methods. To perform the test, pure M. tuberculosis isolates were obtained by culturing an aliquot of 100 μl from one of the control wells on plates containing 7H11 medium (Fisher Scientific) enriched with OADC. The plates were incubated at 37°C for 3 weeks (maximum, 21 days).

For MGIT-PZA, an M. tuberculosis suspension with a turbidity of 0.5 McFarland was prepared using physiological saline. The suspension was diluted to 1:5 and 1:10, and 0.5-ml portions of each dilution were inoculated into the test and the control tubes, respectively. PZA was added to the test tube to a final concentration of 100 μg/ml. The tubes were incubated in a MGIT 960 system. PZA susceptibility was determined by comparing the fluorescent signal between the control and the test tube over a period of 4 to 21 days.

For *pncA* sequencing, one full loop of a 21-day M. tuberculosis culture was suspended in 100 mM Tris-EDTA buffer (pH 8.0). The suspension was inactivated by heating to 80°C for 30 min, and genomic DNA was extracted after a modified proteinase K-chloroform protocol (58). Briefly, 100 μl of 10 mg/ml of lysozyme was added, and the sample was incubated overnight at 37°C in agitation. The following day for sample digestion, 20 mg/ml of proteinase K and 10% sodium dodecyl sulfate was added, and the sample was incubated at 65°C for 3 h. After that, 5 M NaCl–10% CTAB (cetyltrimethylammonium bromide) was added, and the sample was incubated at 65°C for another 15 min. Then, a solution of phenol-chloroform-isoamilic alcohol (25:24:1) was added in equal volumes, and this was mixed gently. The sample was centrifuged at 10,000 rpm at 4°C for 10 min. The upper layer was transferred to a new tube, and phenol-chloroform was added (1:1). The sample was centrifuged at 10,000 rpm at 4°C for 10 min. This step was repeated. To precipitate the DNA, 95% ethanol was added, and the sample was incubated at –80°C for 30 min. The sample was centrifuged at 10,000 rpm at 4°C for 10 min, and a wash step was performed with 70% ethanol; the pellet was then dried at room temperature. For resuspension, 50 μl of Tris-EDTA buffer (pH 8.0) was added. PCR was performed using the primers P1 (5′-GTCGGTCATGTTCGCATCG-3′; from −105 bp upstream of *pncA*) and P6 (5′-GCTTTGCGGCGAGCGCTCCCA-3′; from 60 bp downstream of *pncA*) (59). The PCR cycling parameters were as follows: one cycle of 94°C for 4 min; followed by 30 cycles of 94°C for 45 s, 58°C for 60 s, and 72°C for 60 s; with a final step of 94°C for 5 min. The PCR product length was 720 bp. Sequencing was performed using the same pair of primers. The M. tuberculosis H37Rv reference strain (NC_000962.3) was used as a reference for the pairwise sequence alignment in order to detect mutations in the *pncA* gene and its putative promoter. PZA susceptibility for the mutations detected was assigned according to the Tuberculosis Drug Resistance Mutation Database (60).

The classic Wayne test was performed as previously described (61). Briefly, three full loops of M. tuberculosis 21-day culture were inoculated in tubes containing Dubos medium, followed by incubation at 37°C for 7 days. Positive (H37Rv) and negative (DM097) controls were included. At day 7, 1 ml of 1% ferrous ammonium sulfate (SAF; Sigma-Aldrich) was added. After 5 min of incubation at room temperature, each tube was read. A positive result (i.e., a PZA-susceptible strain) was reported when a pink band was observed at the top of the media. In contrast, a negative result (i.e., a PZA-resistant strain) was reported when there was an absence of the pink band.

### Data analysis.

The sensitivity and specificity (95% CI) of MODS-PZA was calculated by comparison with the CRT in a 2 × 2 contingency table for each PZA concentration and plate reading day. McNemar’s test was used to determine significant differences between the sensitivities and specificities calculated. The Fisher exact test was used to determine the association between growth level in culture and the determined PZA susceptibility. Agreement between the dichotomous MODS-PZA, the CRT, and MGIT-PZA was estimated by using the kappa statistic. All statistical tests were reported with 5% significance using STATA v14.0.

## RESULTS

### Study population.

A total of 183 sputum samples were collected. One was excluded from the analysis due to inconsistent results. Of the remaining 182 samples, 51.1% (93/182) were reported as RIF and INH susceptible, 30.22% (55/182) were MDR-TB (i.e., resistant to both RIF and INH), 12.09% (22/182) were monoresistant to INH, and 6.59% (12/182) were monoresistant to RIF. Data regarding patient age and sex, site of sample collection, tuberculosis treatment, and sputum acid-fast microscopy results are presented in Table 1.

**TABLE 1:** Study population characteristics^*a*^

Variable	No. (%)
Collection center	
Callao Reference Laboratory	140 (76.9)
Hospital Cayetano Heredia	11 (6.0)
Hospital Dos de Mayo	31 (17.0)
	
Age (yr)	
<30	54 (29.7)
30–60	66 (36.3)
>60	62 (34.1)
	
Sex	
Female	52 (28.6)
Male	108 (59.3)
Data not available	22 (12.1)
	
TB treatment	
Previous or ongoing treatment	83 (45.6)
Never treated	19 (10.4)
Data not available	80 (43.9)
	
Sputum acid-fast microscopy result^*b*^	
Negative	11 (6.0)
Paucibacillary	10 (5.5)
1+	38 (20.9)
2+	58 (31.9)
3+	65 (35.7)

a All samples were selected according to the following criteria: a minimum sample volume of 2 ml and a positive sputum acid-fast smear microscopy result (health center results).

b Paucibacillary, 1 to 9 bacilli in 100 fields; 1+, 10 to 99 bacilli in 100 fields; 2+, 1 to 10 bacilli per field in 50 fields; 3+, 10 bacilli per field in 20 fields.

### Consensus reference test for PZA susceptibility determination.

According to the CRT, 78.57% (143/182) of the samples were reported as PZA susceptible, and 21.43% (39/182) were reported as PZA resistant. A total of 50.91% (28/55) of the MDR-TB samples and 58.33% (7/12) of the RIF monoresistant samples were PZA resistant (Table 2).

**TABLE 2:** Sample distribution according to its PZA and TB resistance profile^*a*^

PZA susceptibility^*b*^	No. (%) with TB various resistance profiles^*c*^				Total no. (%) of samples
	MDR-TB	RIF^r^	INH^r^	RIF^s^ and INH^s^	
PZA susceptible	27 (49.09)	5 (41.67)	21 (95.45)	90 (96.77)	143 (78.57)
PZA resistant	28 (50.91)	7 (58.33)	1 (4.55)	3 (3.23)	39 (21.43)

a Results were determined by a consensus reference test (CRT), comprising MGIT, Wayne assay, and *pncA* sequencing, and MODS culture, respectively. PZA susceptibility is displayed according to the TB susceptibility profile.

b As determined by a CRT.

c RIF^r^, RIF (rifampin) monoresistant; INH^r^, isoniazid (INH) monoresistant; RIF^s^, RIF susceptible; INH^s^, INH susceptible.

According to MGIT-PZA, 75.27% (137/182) of the samples were reported as PZA susceptible, and 24.73% (45/182) as PZA resistant. Six samples did not agree with the CRT. Four of these discordant samples had the K48T and F81S *pncA* mutations, which have been reported as showing a PZA-resistant phenotype by MGIT-PZA when this method was performed using 100 μg/ml of PZA. However, a higher critical concentration of PZA (300 μg/ml) classified these isolates as PZA susceptible (data not shown) in concordance with the classical Wayne test and *pncA* sequencing. The other two presented a wild-type genotype.

According to the classic Wayne test, 83.52% (152/182) of the samples were PZA susceptible and 16.48% (30/182) were PZA resistant. Nine samples showed discordancy with the CRT; all nine samples had mutations detected in the *pncA* gene (H51R, Q10P, and Q10R).

Finally, by *pncA* sequencing, 26.92% (49/182) of the samples reported a nonsynonymous *pncA* mutation (i.e., in its putative promoter or in the gene itself), and 73.08% (133/182) displayed the wild-type genotype. Q10R was the most frequently observed mutation (28.57%), followed by H51R (18.37%), and K48T (16.33%). All other reported mutations were found in fewer than five samples, including one sample with the A-11C (nucleotide) mutation in the *pncA* promoter (Table 3). The P62S, K48T, and F81S mutations (i.e., observed in this study) were linked to a PZA-susceptible phenotype according to the Tuberculosis Drug Resistance Mutation Database (54). Overall, 79.12% (144/182) and 20.88% (38/182) of samples were reported as PZA susceptible and resistant, respectively. Just one sample was discordant with the CRT, in which *pncA* sequencing did not report any mutations, but both MGIT-PZA and the classic Wayne test were reported as showing PZA resistance (see Table S1 in the supplemental material).

**TABLE 3:** *pncA* mutations reported in this study^*a*^

Genotype	No. (%) of isolates^*b*^	PZA susceptibility (database/literature)^*c*^	PZA susceptibility (CRT)^*d*^
D49N	1 (0.55)	Resistant	Resistant
D8E	2 (1.10)	Resistant	Resistant
F81S	2 (1.10)	Susceptible	Susceptible
H51R	9 (4.95)	Resistant	Resistant
H57L	1 (0.55)	Resistant	Resistant
H71R	2 (1.10)	Resistant	Resistant
I6S	2 (1.10)	Resistant	Resistant
K48T	8 (4.40)	Susceptible	Susceptible
P62S	1 (0.55)	Susceptible	Susceptible
A-11G	1 (0.55)	Resistant	Resistant
Q10P	2 (1.10)	Resistant	Resistant
Q10R	14 (7.69)	Resistant	Resistant
Δ375–389	1 (0.55)	Resistant	Resistant
Δ456–466	3 (1.65)	Resistant	Resistant
Wild type	133 (73.08)	Susceptible	Susceptible

a Mutations observed among the putative promoter and *pncA* gene are shown, along with their corresponding PZA susceptibility phenotype, as defined by both the Tuberculosis Drug Resistance Mutation Database and our consensus reference test (CRT).

b The percentage was calculated based on the total number of samples (*n* = 182).

c According to the Tuberculosis Drug Resistance Mutation Database and the literature.

d As determined by CRT (MGIT, Wayne assay, and *pncA* sequencing).

### Growth level in culture.

M. tuberculosis culture growth was classified into four growth levels: high, regular, low, and microcolonies only. Ninety-three percent of the samples reported at least a low level of growth in culture, including 94.4% of the PZA-sensitive isolates (determined by the CRT) and 89.74% of the PZA-resistant isolates (Table 4). No significant association (*P* = 0.107) was found between the level of growth on culture and PZA susceptibility, as determined by CRT.

**TABLE 4:** Level of mycobacterial growth in the control well of the MODS-PZA reported by PZA susceptible profile^*a*^

PZA susceptibility^*b*^	Growth level expressed as no. (%) in control wells in a MODS-PZA assay				
	Microcolonies	Low	Regular	High	Total
Susceptible	8 (5.59)	13 (9.09)	50 (34.97)	72 (50.35)	143 (100)
Resistant	4 (10.26)	8 (20.51)	9 (23.08)	18 (46.15)	39 (100)
Total	12 (6.59)	21 (11.54)	59 (32.42)	90 (49.45)	182 (100)

a Four levels of growth were determined according to microscopic evaluation: “Microcolonies” indicates fewer than 20 microcolonies in the well, “low” refers to a barely covered well, “regular” indicates half of the well was covered, and “high” indicates a completely covered well. No significant association (*P* > 0.05) was found between PZA susceptibility and the level of growth.

b As determined by CRT.

### MODS-PZA performance.

A similar percentage of PZA-susceptible and -resistant samples was observed in both PZA wells (400 and 800 μg/ml) at the first plate reading (R1). Twelve samples showed a conversion to PZA resistance at 400 μg/ml PZA for the second plate reading (R2), and eight samples showed a conversion to PZA resistance at 800 μg/ml. For R1, three samples showed discordant results at between 400 and 800 μg/ml PZA. One of these samples presented a wild-type genotype and was determined to be PZA susceptible by the CRT. The remaining two samples were PZA resistant, with nucleotide deletions in the *pncA* gene. Likewise, for R2 seven samples showed discordant results. Three samples were determined to be PZA resistant by the CRT, and all of these had the Q10R mutation and a deletion in the *pncA* gene. The other four samples were determined to be PZA susceptible according to the CRT, and just one had the F81S mutation. No significant association was observed between PZA susceptibility determined in R1 and the level of growth using either 400 μg/ml (*P* = 0.069) or 800 μg/ml (*P* = 0.064) PZA. However, a significant association was reported for PZA susceptibility in R2 at both 400 μg/ml and 800 μg/ml PZA (*P* = 0.045 and *P* = 0.021, respectively). Although not significant, an association was observed for R1 at both concentrations, with a tendency toward an increase (approximately 40 to 50%) for a high growth level for PZA-resistant isolates from R1 to R2 in both concentrations. This event did not occur for PZA-susceptible isolates, for which the high growth level percentage was around 50% at both R1 and R2. The percentages for the other growth levels did not show any changes from R1 to R2 (data not shown).

### Accuracy measures for MODS-PZA.

Results obtained at each PZA concentration and reading day were compared independently to the CRT in order to evaluate sensitivity and specificity. For both drug concentrations, the sensitivity and specificity were >70 and >90%, respectively (Table 5). When using a PZA concentration of 400 μg/ml, a significant difference was observed between R1 and R2 in specificity (*P* = 0.016) but not in their sensitivity (*P* = 0.063). On the other hand, no significant difference in sensitivity or specificity was observed when using PZA concentrations of 800 μg/ml between R1 and R2 (both *P* = 0.125). In addition, no significant difference was observed in sensitivity and specificity between 400 and 800 μg/ml of PZA in R1 (*P* = 0.5 and *P* = 1.0, respectively) and in R2 (*P* = 0.25 and *P* = 0.125, respectively). A kappa index and agreement percentage of 0.79 and 93.41%, respectively, were reported for a PZA concentration of 400 μg/ml, and 0.77 and 92.86%, respectively, for a PZA concentration of 800 μg/ml; both in R1. Similarly, in R2 a kappa index and agreement percentage of 0.78 and 92.31%, respectively, were reported for 400 μg/ml PZA, and 0.79 and 92.86%, respectively, for 800 μg/ml PZA.

**TABLE 5:** MODS-PZA performance was evaluated against the consensus reference test^*a*^

PZA dose (μg/ml)	Plate reading day	Sensitivity		Specificity		Kappa index	Agreement (%)
		% (*n/N*)	95% CI	% (*n/N*)	95% CI		
400	R1	76.9 (30/39)	0.61–0.89	97.9 (140/143)	0.94–0.99	0.79	93.4
	R2	89.7 (35/39)	0.76–0.97	93.0 (133/143)	0.88–97.00	0.78	92.3
800	R1	71.8 (28/39)	0.55–0.85	98.6 (141/143)	0.95–0.99	0.77	92.8
	R2	82.1 (32/39)	0.66–0.92	95.8 (137/143)	0.91–0.99	0.79	92.9

a Each accuracy measurement was calculated with a 95% confidence interval (95% CI). R1, first plate reading; R2, second plate reading. *n/N*, number positive/total number tested.

From the samples that had discordant results for MODS-PZA at 400 μg/ml PZA and the CRT, those that were reported as PZA resistant by MODS-PZA but PZA susceptible by the CRT showed either a wild-type genotype or the P62S or F81S mutations in the *pncA* gene. However, these mutations have been previously reported in the PZA-resistant phenotype associated with mutations represented in a M. tuberculosis mutant *pncA* library, where the PZA susceptibility was detected by MGIT 960 and bioinformatics prediction (62). On the other hand, samples that were reported to be PZA susceptible by MODS-PZA but PZA resistant by CRT had Q10R, Q10P, H51R, H71R, and A-11C mutations detected in the *pncA* gene and its putative promoter. All of these mutations have been reported to confer PZA resistance with high confidence (60, 63). No differences between bacillary load (i.e., by sputum acid-fast smear or level of growth in culture) were observed (data not shown). The same results were observed for isolates with discordancy between MODS-PZA performed at 800 μg/ml PZA and the CRT, including samples in which *pncA* deletions (nucleotide deletions Δ375–389 and Δ456–466) were detected and yet were reported as PZA susceptible in 800 μg/ml of PZA.

MODS-PZA was compared to MGIT-PZA since both tests are based on the principle that culture in the presence of the drug would inhibit bacterial growth. A kappa index of 0.74 and an agreement percentage of 91.21% were found for 400 μg/ml at R1, a kappa index of 0.72 and agreement of 90.66% were found for 800 μg/ml at R1, a kappa index of 0.79 and agreement of 92.31% for 400 μg/ml were found at R2, and finally a kappa index of 0.77 and an agreement of 91.76% for 800 μg/ml were found at R2. The median turnaround time for MODS-PZA was 10 days (range, 5 to 24 days) for R1, and the R2 was performed a median of 3.1 days after R1.

## DISCUSSION

This study reports, for the first time, the application of MODS culture to determine PZA resistance directly from sputum culture through the evaluation of PZA activity at neutral pH (MODS-PZA). Unlike other studies that have evaluated the application of MODS for PZA resistance determination in M. tuberculosis strains (46–48), our findings have demonstrated the possibility of directly evaluating sputum samples with high PZA concentration (400 and 800 μg/ml) without the need for additional processing except for a decontamination step. Including PZA in the spectrum of M. tuberculosis drugs that are currently tested in MODS (RIF, INH, and second-line drugs) (41–43, 64) will give a complete drug susceptibility test that could be applied routinely in low-income countries.

The principal limitation for traditional phenotypic DST for PZA susceptibility determination has been the requirement of an acidic pH in order to allow PZA to show antituberculosis activity (15, 29, 30). *In vivo*, a concentration peak in blood between 20 and 60 μg/ml (1 to 2 h postdose) is active against M. tuberculosis (65). These concentrations are active during the early phase of infection because the physiological pH is reduced due to ongoing inflammatory processes. According to the proposed mechanism of action for PZA, when POA is released in an acidic medium (pH 5.5), some molecules are protonated (due to its pKa of 2.9) and reenter the cytoplasm, showing its bactericidal effect (66). *In vitro*, a similar condition can be obtained when the culture is performed at pH 5.5 (using an MIC of 50 μg/ml). However, an acidic pH has been reported to inhibit M. tuberculosis growth (30, 31). Theoretically, higher concentrations of PZA can show antituberculosis activity at a higher pH. Therefore, an active PZA concentration can be estimated for a particular pH using the Henderson-Hasselbach equation (67). One disadvantage of this methodology is that susceptibility results of DST using higher drug concentrations cannot be directly related to pharmacokinetic parameters determined *in vivo*. However, it should be considered that the objective of any DST is the discrimination between susceptible and resistant strains. For this study, PZA concentrations of 400 and 800 μg/ml were selected to evaluate PZA susceptibility based on the expected active PZA concentration for a pH of 6.8 (estimated concentration, 992 μg/ml) and some past studies that reported a PZA activity of 300 to 400 μg/ml in 7H12 broth at pH 6.0 to 6.2 (68–70).

Even though no significant differences were reported between the sensitivities of the two reading time points (R1 and R2) in this study (*P* > 0.05), reported that sensitivities were lower than those shown in other studies. All other studies (46–48) have reported a sensitivity close to 95% but used 100 μg/ml PZA as the critical concentration and at a lower pH (6.0); although these studies evaluated higher concentrations of PZA, ranging from 6.25 to 3,200 μg/ml, 100 μg/ml was used as the cutoff for sensitivity determination. However, this concentration has been shown to lead to the false reporting of isolates as resistant (36, 39, 68) since it is lower than the expected PZA concentration for pH 6.0. It is important to bear in mind that none of those studies used a CRT as a gold standard: they compared MODS for PZA against MGIT-PZA (46), the broth microdilution method (47), and the proportion method (48).

Another important difference that may account for the observed sensitivity variation is that those studies used a McFarland dilution of M. tuberculosis strains previously isolated in Lowenstein-Jensen medium as the inoculum, rather than direct inoculation of sputum samples. This could therefore indicate that a larger number of viable mycobacteria were evaluated than would be found in clinical samples. The specificity, which ranged from 93.0 to 98.6%, was similar to that reported in other studies close to 95% (46, 48); Ghiraldi et al. (47) reported a sensitivity of 100%, but the number of PZA-resistant strains included in that study was small (*n* = 8).

Discordant samples that were reported as PZA sensitive by MODS-PZA but PZA resistant by CRT showed mutations in the *pncA* gene or the putative promoter, independently of the concentration of PZA. The mutations observed in these isolates were H51R, H71R, Q10P, Q10R, A-11C, Δ375–389, and Δ456–466. All of these mutations have been strongly associated with PZA-resistant phenotypes (11, 22, 25, 63). These mutations affect the catalytic site or the metal-binding site of PZAse, eliminating its enzymatic activity (11, 22, 63). Almost all of these isolates were MDR-TB or monoresistant to RIF (88.89% for 400 μg/ml and 90.91% for 800 μg/ml PZA in R1 and 100% for both 400 and 800 μg/ml PZA in R2) and demonstrated at least a low level of growth in control wells. On the other hand, discordant samples that were reported as PZA resistant by MODS-PZA but PZA sensitive by CRT showed a wild-type genotype or carried the F81S or P62S mutations, which are reported as being associated with PZA-sensitive phenotypes in the Tuberculosis Drug Resistance Mutation Database (60). These isolates were MDR-TB, monoresistant to INH, or non-MDR-TB. The growth level in culture did not show the same tendency as the previously described discordant samples: these samples were reported as either microcolonies only or having a high growth level. Although no plausible explanation was found for these observations, some similar discordances have been reported elsewhere (48). PZA-sensitive strains with a wild-type genotype that were reported as PZA resistant by MODS showed a PZA-sensitive phenotype by the proportion method in Lowenstein-Jensen medium, with an MIC ranging from 600 to 1,200 μg/ml. Likewise, a PZA-sensitive strain with the L35P mutation (associated with a PZA-sensitive phenotype) was reported as PZA resistant in MODS with an MIC of 1,200 μg/ml PZA.

Upon comparison to MGIT-PZA, since both assays have the same rationale, the test showed a sensitivities ranging from 68.9 to 84.4% for R1 and from 64.4 to 75.6% for R2 for 400 and 800 μg/ml PZA, respectively. The specificity fell within a narrower range of 94.9 to 98.7%, with an agreement close to 91.0%. However, it must be noted that MGIT-PZA is performed with M. tuberculosis cultures. A previous study (71) that evaluated the performance of MGIT-PZA directly from sputum samples reported uninterpretable results for 163 samples, varying from 23 to 66% among all of the participating laboratories. Moreover, the percentage of successful MGIT-PZA procedures performed directly from sputum samples compared to traditional MGIT-PZA was 59% (range, 34 to 77%). Therefore, MODS-PZA could be comparable to the “direct MGIT-PZA” method. Median turnaround time of MODS-PZA were 10 and 12 days for PZA-sensitive isolates (range, 5 to 24 days) and PZA-resistant isolates (range, 5 to 21 days), respectively; the traditional MGIT-PZA reports a similar turnaround time ranging from 4 to 21 days (71).

It is important to mention that it remains vital to define the exact mechanism of action of PZA in order to develop the most suitable susceptibility test for this drug, which is considered the pivot in most current and future treatments. More data are needed to increase our understanding of the synergy between different factors that can enhance or reduce PZA activity under controlled *in vitro* conditions.

This is the first time that MODS culture has been adapted to evaluate PZA susceptibility directly from sputum samples, obtaining a relatively inexpensive, simple, and fast phenotypic DST. Currently, the implementation of any MODS based DSTs (such as MODS-PZA) will have limitations related to laboratory infrastructure (MODS requires biosafety level 2 facilities) (72), equipment acquisition (inverted microscope), and personnel training in the interpretation of results. However, some advantages of the MODS test should be highlighted. Compared to other commercial cultures or LPA-based assays, MODS is less expensive (approximately U.S. $4 to $5 compared to U.S. $15 to $56, not considering any additional equipment) (73, 74). Although there is no considerable cost difference compared to gene sequencing (US $8 to $12), in limited-resource countries this procedure is usually outsourced, which implies an additional and expensive shipping service (28). Regarding the infrastructure and equipment implementation, every phenotypic DST has similar minimum requirements with the exception of automatized tests (i.e., MGIT-PZA) which increase the total cost of the assay due to the acquisition of the specific instrument. Although molecular tests (such as LPA-based assays) may not require additional expensive instrumentation, they face an important downside since not all *pncA* mutations have been reported and associated with PZA resistance (26, 27). Consequently, growth-based tests are still considered the standard test (or at least a complementary test) for PZA susceptibility determination (27, 39).

Recently, our group have reported an adaptation of the classic Wayne test on MODS culture directly from sputum samples (75). This test permits an indirect determination of PZA resistance by the detection of POA produced at pH 7.0 (i.e., with a sensitivity and specificity of 92.7 and 99.3%, respectively) without the necessity for a primary culture. However, one critical point of this test is the addition of PZA and ferrous ammonium sulfate. For this, PZA is added after M. tuberculosis growth for an average of 10 days and then, after three more incubation days, ferrous ammonium sulfate is added in order to determine PZA susceptibility phenotype. As a result, the culture plate must be opened twice when M. tuberculosis has just shown growth. This means that the risk of cross contamination or culture spillover could increase if technicians do not work very carefully. Thus, MODS-PZA is an alternative direct phenotypic DST which does not require the addition of any reagent or the manipulation of the plate, other than for microscopic evaluation (i.e., the culture plate is never opened after the sputum sample inoculation).

In spite of the sensitivity and specificity values achieved in this study, more work is needed to evaluate the effect of other stress factors on MODS-PZA, since adjusting for these may enable a further increase in its accuracy. The final goal is the development of a complete, reliable, fast, and low-cost PZA susceptibility assay that could reduce the rate of nondiagnosed PZA resistance cases, especially in low-setting countries where TB is still an endemic disease.

## Supplementary Material

Supplemental file 1
